# Recombination fraction and genetic linkage among key disease resistance genes (*Co-4^2^*/*Phg-2 and Co-5*/“P.ult”) in common bean

**DOI:** 10.5897/AJB2019.16776

**Published:** 2019-09-30

**Authors:** Dennis Okii, Arfang Badji, Thomas Odong, Herbert Talwana, Phinehas Tukamuhabwa, Allan Male, Clare Mukankusi, Paul Gepts

**Affiliations:** 1 Department of Agricultural Production, Makerere University, P. O. Box 7062, Kampala, Uganda; 2 International Centre for Tropical Agriculture (CIAT)/Pan African Bean Research Alliance (PABRA), P. O. Box 6247, Kampala, Uganda; 3 Section of Crop and Ecosystem Sciences, Department of Plant Sciences/MS1, University of California, 1 Shields Avenue, Davis, CA 95616-8780, USA

**Keywords:** Genetic linkage, recombination, likelihood, logarithm of odds (LOD) score, sequence characterized amplified regions (SCAR) markers, genes

## Abstract

Anthracnose (*Colletotrichum lindemuthianum*), Angular leaf spot (*Pseudocercospora griseola*) and *Pythium* root rot are important pathogens affecting common bean production in the tropics. A promising strategy to manage these diseases consists of combining several resistance (R) genes into one cultivar. The aim of the study was to determine genetic linkage between gene pairs, *Co-4^2^*/*Phg-2*, on bean-chromosome Pv08 and *Co-5*/“P.ult” on-chromosome Pv07, to increase the efficiency of dual selection of resistance genes for major bean diseases, with molecular markers. The level of recombination was determined by tracking molecular markers for both BC3F6 and F2 generations. Recombination fraction r, among gene pairs, the likelihood of linkage, L(r), and logarithm of odds (LOD) scores were computed using the statistical relationship of likelihood which assumes a binomial distribution. The SCAR marker pair SAB3/PYAA19 for the gene pair *Co-5*/“P.ult” exhibited moderate linkage (r = 32 cM with a high LOD score of 9.2) for BC_3_F_6_ population, but relatively stronger linkage for the F_2_ population (r = 21 cM with a high LOD score of 18.7). However, the linkage among SCAR marker pair SH18/SN02, for the gene pair *Co-4^2^*/*Phg-*2 was incomplete for BC_3_F_6_ population (r = 47 cM with a low LOD score of 0.16) as well as F_2_ population (r = 44 cM with a low LOD score of 0.7). Generally, the weak or incomplete genetic linkage between marker pairs studied showed that all the four genes mentioned earlier have to be tagged with a corresponding linked marker during selection. The approaches used in this study will contribute to two loci linkage mapping techniques in segregating plant populations.

## INTRODUCTION

Diseases are critical production constraints for common beans in tropical countries, causing total crop failure when several pathogens attack susceptible bean genotypes under favorable conditions (Schwartz and Galvez, 1980; Mahuku et al., 2011). Management of diseases at farm level in short and long term is practical, through genetic control by incorporating resistant alleles for different pathogens into selected cultivars through marker assisted gene pyramiding techniques (Young and Kelly, 1996; Malav et al., 2016).

Marker assisted breeding strategies for pyramiding genes requires information on marker co-transmissions (Mahuku et al., 2011; http://www.extension.org/pages/32465/gene-pyramidingusing-molecularmarkers#.ViTJovlViko). Molecular markers and genetic maps accelerate identification of desirable homozygotes without need for progeny tests (Pathania et al., 2014).

Genetic maps and markers of major disease of beans are reported for bean breeding (Kelly et al., 2003; Oblessuc et al., 2013; Perseguini et al., 2016) and in the Phaseolus Genes database (http://phaseolusgenes.bioinformatics.ucdavis.edu/; Miller et al., 2018) and may be utilised in gene pyramiding.

Generating breeding information on linkages of sequence characterized amplified region (SCAR) marker pairs and their co-segregations could help to reduce sample sizes and time during marker assisted pyramiding (stacking together) of major genes in breeding programs for managing multiple diseases of common bean.

Practically, a polymorphic marker must co-segregate with the gene of interest and so be present in the resistant progeny lines but absent in susceptible ones (Miller et al., 2018). Once linkage is established between markers, the chromosomal region flanking the marker can then be analyzed for alternative markers.

Genetic linkage is the tendency of genes that are located proximally to each other on a chromosome to be inherited together during meiosis and can therefore be used as a tool for estimating the genetic distance between two loci (Ott et al., 2015). Two statistical approaches, termed parametric and nonparametric linkage analysis have been used to test, linkage analysis or observed recombination between two loci and is detailed by Bailey-Wilson and Wilson (2011).

Parametric linkage analysis was applied in this study and its test statistic is called the logarithm of odds (LOD) score (Balding et al., 2007; Strachan and Read, 2011). A LOD score higher than 3.0 is generally accepted as the evidence supporting linkage, whereas a LOD score lower than -2.0 is considered evidence against linkage (Ott et al., 2015).

LOD score analysis is a simple way to determine the linkage between Mendelian traits (or between a trait and a marker, or two markers). On the other hand, the nonparametric linkage analysis is a model-free approach that studies the probability of an allele being identical by descent. Balding et al. (2007) and Strachan and Read (2011) describe the LOD score method in greater detail. Briefly, it works as follows: estimates of recombinant and non-recombinant fraction is made, the overall likelihood, given linkage and the likelihood, given no linkage and a LOD score is calculated for each estimate of recombination fraction.

The recombination fraction estimate with the highest LOD score is considered the best estimate. The two-point LOD score between two loci, that is, a trait and a marker or marker-marker loci in this study was typically calculated over several recombination fractions between 0 and 1/2, and the recombination fraction that maximizes the likelihood (the maximum LOD score) is considered to be the best estimate of the recombination fraction (Bailey-Wilson and Wilson, 2011).

For most occurrences of crossing-over, genes located at close physical postion are co-inherited due to linkage, while genes far apart tend to segregate independently (Ott et al., 2015). Genetic recombination through chromosomal cross-over after hybridization produces new haplotypes during meiosis through interchromosomal genetic material exchange and plays a critical role in the evolution of organisms (Coop and Przeworski, 2007).

Estimates of recombination rates are traditionally obtained by directly counting the number of such events during meiosis (Kaplan and Hudson, 1985). This approach is however, limited by the extremely low fraction of recombinations between tightly linked genes (Gao et al., 2016). Two loci that are far apart on the chromosome have a high probability of recombination in any meiosis, such that they assort independently to offspring (Bailey-Wilson and Wilson, 2011). While, loci that are very far apart experience recombination about 50% of the time, and thus appear to be assorting independently, just as loci on different chromosomes do. Probability refers to knowing parameters (measurable characteristic of a system) and being able to predict their outcomes, while Likelihood is a synonym for probability where observed data is used to estimate parameters (Edwards, 1972). The likelihood ratio test that is the basis of modern parametric likelihood ratio tests for linkage (Bailey-Wilson and Wilson, 2011) likelihood ratio test that is the basis of modern parametric likelihood ratio tests for linkage (Bailey-Wilson and Wilson, 2011). In this study, the likelihood hypothesing linkage was compared to a hypothesis of no linkage with some specific recombination fraction (r < 1/2).

At data analysis levels, observations are already completed, the data is fixed and there is no probabilistic part of the data anymore (Edwards, 1972). Likelihood of the model parameters that underlie the fixed data would then be of most interest. Maximum likelihood estimation (MLE) thus aims to find the parameter value(s) that makes the observed data most likely (Staub et al., 1996; Toomet and Henningsen, 2009). This study aimed at estimating recombination frequencies and genetic linkage between gene pairs, *Co-4^2^/Phg-2* on bean chromosome Pv08 and *Co-5*/“P.ult” on chromosome Pv07.

In the study, the gene symbol “P.ult” is for *Pythium ultimum* root rot resistance gene linked to (SCAR) marker PYAA19 developed by Mahuku et al. (2007) for selecting bean lines resistant to several species of *Pythium* root rot in common bean. According to the developers of the SCAR marker, the linked gene symbol is *Pyult1*. The gene symbol for root rot disease used in this study is “P.ult”, shortened from the targeted pathogen’s genus (*Pythium)* and species (*ultimum)* names. The gene symbol (“P.ult”) was thus put between quotation marks within the text and not italicised, unlike the other genes in this study (that is, *Co-4^2^* , *Phg-*2 and *Co-5*) because it is not an official genetic symbol in common beans.

The findings will contribute to the efficiency of marker assisted pyramiding of disease resistance genes in common bean leading to simultaneous expression of more than one gene in a variety to develop durable resistance expression (Malav et al., 2016).

## MATERIALS AND METHODS

### Population development

**Figure 1:**
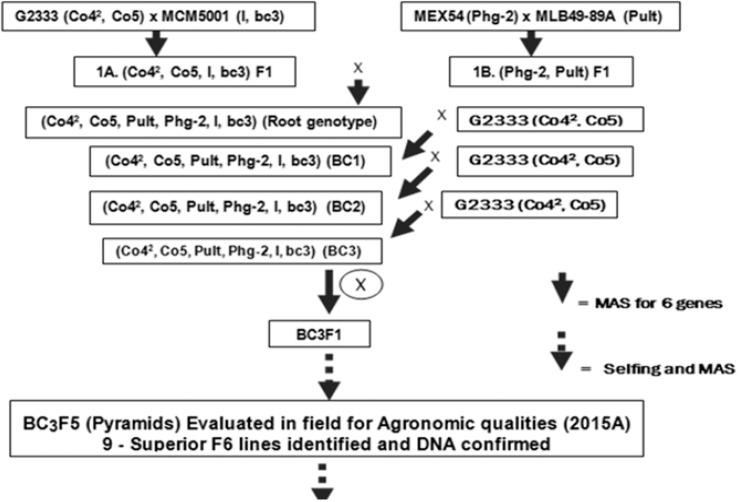
Simultaneous gene pyramiding scheme developed and used via MAS.

**Figure 2:**
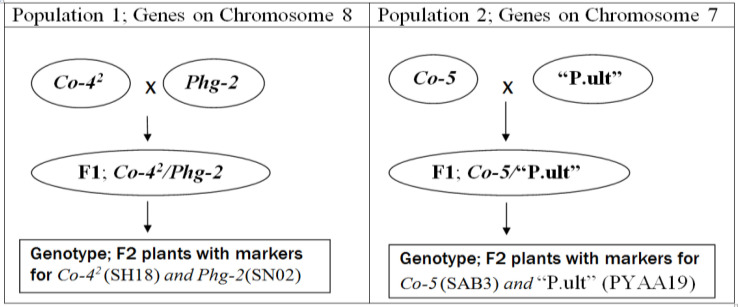
Scheme used to develop populations for testing linkage among genes physically located on bean chromosomes eight and seven.

Two segregating bean populations: BC_3_F_6_ andF_2_ were developed for this study and are described as follows. In the start, a BC_3_F_5_ population previously developed at CIAT of Kawanda (Uganda) to combine six disease resistance genes of common bean, namely *Co-4^2^, Co-5, Phg-2,* “P.ult”*, I* and *bc-3* (Figure 1) formed the genetic material for this study. The BC_3_F_5_ populationwas plantedinthe fields at CIAT, Kawanda in 2015 to advance it to BC_3_F_6_ population from which DNA of 345 plants was collected and genotyped with SCAR markers in the molecular laboratory facility at CIAT, Kawanda as detailed subsequently. Bean plants with single dorminant genes (specifically, *Co-4^2^, Co-5, Phg-2,* “P.ult”*)* were identified among the 345 plants of BC_3_F_6_ population using genotypic electrophoresis gel profiles, harvested and seed used as parents to develop new crosses and populations (Figure 2) in the screen house facility at CIAT, Kawanda.

Five representative plants from progenies of the BC_3_F_6_ population with single genes were planted in 5 litre's plastic pots with sterile soils, labelled and monitored with daily watering until flowering and crosses made through hand pollinations to generate two F1 populations between respective plants with targeted single genes (*Co-4^2^ x Phg-2 – population 1* and *Co-5 x* “P.ult” *– population 2*). The F1 seeds from the two populations were harvested separatelty dried and planted in the screen house in the second season in 2015 to generate two populations of F2 plants which were monitored until maturity and harvested. The F2 seeds were planted in the screen house in sterile soils in raised wooden trays measuring 75 cm long × 45 cm wide and height of 13 cm during the third season to generate plants for DNA extraction.

### DNA extraction

The genomic DNA from 345 plants from BC_3_F_6_ population in the field was collected from young leaves before flowering in eppendorf tubes, clearly labelled and transferred to the laboratory for extraction. DNA of each plant was isolated in the molecular laboratory facility at CIAT, Uganda, using the CTAB method according to Mahuku (2004), and kept in Eppendorf tubes at -20°C for further analyses.

The two F2 populations having 219 plants targeting genes; *Co-4^2^ x Phg-2* – population 1 and 236 plants targeting Co-5 x “P.ult” - population 2 were planted in trays in the screen house and DNA was extracted from seedlings at the second trifoliate stage. The DNA was extracted using the Whatman FTA card technology according to Chilagane et al., (2013). The leaf sample was placed over the marked area of the FTA Plant Saver card and the leaf was overlaid with parafilm. The leaf was pounded using a pestle, making sure that the leaf material was transferred to the paper by checking the back of the FTA card.

The samples were left to air dry and later transferred to the laboratory. The Harris 2 mm unicore punch, was used to cut the samples from the FTA cards with the assistance of the cutting mat and placed into the PCR tube and then washed twice using 200 ml of FTA purification reagent followed by 200 ml of 1X TE buffer (10 mM Tris HCl, 0.1 mM EDTA, pH 8.0) and the sample left to incubate at room temperature for about 10 min in each wash, then the leaf disks were left to dry and ready for PCR reaction.

### Polymerase chain reaction (PCR) and electrophoresis

DNA samples for amplification through PCR were diluted to a factor of 1 in 30 µl solution and sorted out according to the field plan. The PCR reaction mix contained 5 µl of the *Accu-Power PCR* premix composed of DNA polymerase, dNTPs, reaction buffer, blue tracking dye and patented stabiliser. One microlitre of DNA for plants from BC_3_F_6_ population and leaf disk for the two F2 populations, 0.3 µl of forward and reverse primer, and 3.4 µl water was added to the premix to make a total reaction volume of 10 µl. The test sample tubes were placed in a thermocycler (MyGenie, Daejeon) for the PCR reaction cycles. Forward (F) and Reverse (R) primers of SCAR marker SBB14 (F-GTGGGACCTGTTCAAGAATAATAC, R-GTGGGACCTGGGTAGTGTAGAAAT) was used to tag common bean lines with the *Co-4^2^* gene, SN02 (FACCAGGGGCATTATGAACAG, R-ACCAGGGGCAACATACTATG) for the *Phg-2* gene, PYAA19 (F-TTAGGCATGTTAATTCACGTTGG, R-TGAGGCGTGTAAGGTCAGAG) for “P.ult” and SAB3 (F-TGGCGCACACATAAGTTCTCACGG, R-TGGCGCACACCATCAAAAAAGGTT) for *Co-5*. The PCR products of markers used were separated on 1.2% agarose gel in 1X TBE at 140V for 30 min. The gel was then stained in 0.5ug/mL ethidium bromide for 20 min and the image was captured using the Syngene G: BOX gel documentation system (Syngene, Fredrick, MD).

## DATA ANALYSIS

### Marker scoring and establishing number of recombinants

The SCAR marker frequencies (SH18 for *Co-4^2^,* SN02 for *Phg-2,* SAB3 for *Co-5* and PYAA19 for “P.ult”) were computed for BC_3_F_6_ andF2 bean populationsto determine the number of recombinants and for further linkage analysis. This involved scoring polymorphic bands observed on electrophoresis gel pictures for each genotype using one for presence and zero for absence of bands. Recombinant genotypes were identified by counting band patterns in gel pictures in both BC_3_F_6_ and F_2_ populations.

### Recombinants, LOD score and likelihood ratio analysis

The methods for estimating LOD score, likelihoods and recombination value were previously described by Geffroy et al., (2008). Recombination fraction, r, among gene pairs, the likelihood L(r) and LOD scores were generated using the following statistical relationship, assuming a binomial distribution of data in MS office Excel.

$$Likelihood\text{, }L(r)=\left(\begin{array}{c} N\\ R \end{array}\right)r^{R}{\left(1-r\right)}^{N-R}$$

where N = total plants genotyped and R = number of recombinants. The likelihood, L(r) of obtaining the aforenetioned data set for recombination rates was computed using the following relationships, for example using data from BC_3_F_6_ population.

$$Likelihood\text{, }L(r)=\left(\begin{array}{c} N\\ R \end{array}\right)r^{R}{\left(1-r\right)}^{N-R}$$

$$\;Constant,\;\left(\begin{array}{c} N\\ R \end{array}\right)=\frac{N!}{R!(N-R)!}$$

$$\text{ Constant }for\text{ Co}4^{2}/\text{Phg}-2=\frac{345!}{\text{162}!(183)!}$$

$$\text{ Constant }for\;Co5/\text{P .ult }=\frac{345!}{112!(233)!}$$

The LOD (log-odds) score is often used to assess the evidence for linkage and according to Balding et al. (2007) is defined by the formula:

$$LOD=log_{10}\left[\frac{L(data|r)}{L\left(data|r=\frac{1}{2}\right)}\right]$$

where r = recombination fraction. *L*(*data* І r) = likelihood value at maximum estimate (MLE), while *L*(*data* І r = ½) = likelihood value at maximum recombination fraction of 0.5.

The two-point LOD score between two loci, that is, markermarker loci was calculated over several recombination fractions between 0 and 1/2, and the recombination fraction that maximizes the likelihood (the maximum LOD score) was considered to be the best estimate of the recombination fraction (Bailey-Wilson and Wilson, 2011).

## RESULTS

### Recombinants, likelihood and LOD scores in BC_3_F_6_ population

The results of likelihoods (r) and LOD scores for testing linkage among gene pairs, *Co-4^2^*/*Phg-2* and *Co-5*/“P.ult” from bean populations at BC_3_F_6_ generation are shown in Table 1. For gene pairs, *Co-4^2^*/*Phg-2*, 162 genotypes were recombinants (having two genes, *Co-4^2^*/*Phg-2*) and the rest of the genotypes (n= 183) were nonrecombinants (Table 1). Similarly, for gene pairs, *Co5*/“P.ult”*, 112* genotypes were recombinants and 233 non-recombinants.

**Table 1:** Likelihoods (r) and LOD scores for testing linkage of *Co-42*/*Phg-2* and *Co-5*/“P.ult” from BC3F6.

Loci	r	r^R^	1-r	(1-r)^N-R^	L_(r)	Lr/L(r=1/2)	LOD score [Log10(Lr/L(r=1/2))]
***Co-42/Phg-2***		**(R=162)**				**(N-R=183)**	
	0.05	1.71E-211	0.95	8.38E-05	2.33E-113	1.03E-111	-111.00
	0.10	1.00E-162	0.90	4.23E-09	6.88E-69	3.03E-67	-66.52
	0.15	3.36E-134	0.85	1.21E-13	6.63E-45	2.92E-43	-42.53
	0.20	5.85E-114	0.80	1.84E-18	1.75E-29	7.72E-28	-27.11
	0.25	2.93E-98	0.75	1.37E-23	6.51E-19	2.87E-17	-16.54
	0.30	1.97E-85	0.70	4.50E-29	1.44E-11	6.33E-10	-9.20
	0.35	1.38E-74	0.65	5.80E-35	1.30E-06	5.72E-05	-4.24
	0.40	3.42E-65	0.60	2.52E-41	0.0014	0.062	-1.21
	0.45	6.61E-57	0.55	3.06E-48	0.033	1.45	0.16
	0.50	1.71E-49	0.50	8.16E-56	0.023	1.00	-1.24E-10
***Co-5/“P.ult”***		**(R=112)**				**(N-R=233)**	
	0.05	1.93E-146	0.95	6.45E-06	1.57E-58	8.90E-48	-47.05
	0.10	1.00E-112	0.90	2.18E-11	2.76E-30	1.56E-19	-18.81
	0.15	5.28E-93	0.85	3.60E-17	2.40E-16	1.36E-05	-4.868
	0.20	5.19E-79	0.80	2.63E-23	1.73E-08	9.79E+02	3.00
	0.25	3.71E-68	0.75	7.75E-30	0.0004	2.06E+07	7.31
	0.30	2.74E-59	0.70	8.10E-37	0.02	1.59E+09	9.20
	0.35	8.62E-52	0.65	2.56E-44	0.02	1.58E+09	9.20
	0.40	2.70E-45	0.60	2.04E-52	0.0007	3.94E+07	7.60
	0.45	1.44E-39	0.55	3.20E-61	5.84E-07	3.31E+04	4.52
	0.50	1.93E-34	0.50	7.24E-71	1.76E-11	1.00E+00	2.08E-07

### Graphics of likelihood, recombination rates and LOD score in BC_3_F_6_ population

**Figure 3:**
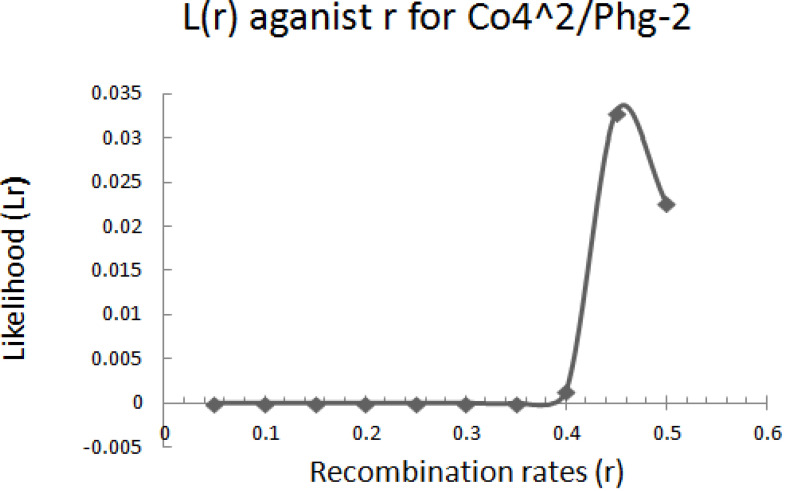
Plot of Likelihood (r) against Recombination rates, showing maximum likelihood estimate (MLE) of recombination fraction at 0.47, so that is the best estimate of linkage for gene pairs *Co-4^2^/Phg-2* on bean chromosome Pv08, using BC3F6 population.

**Figure 4:**
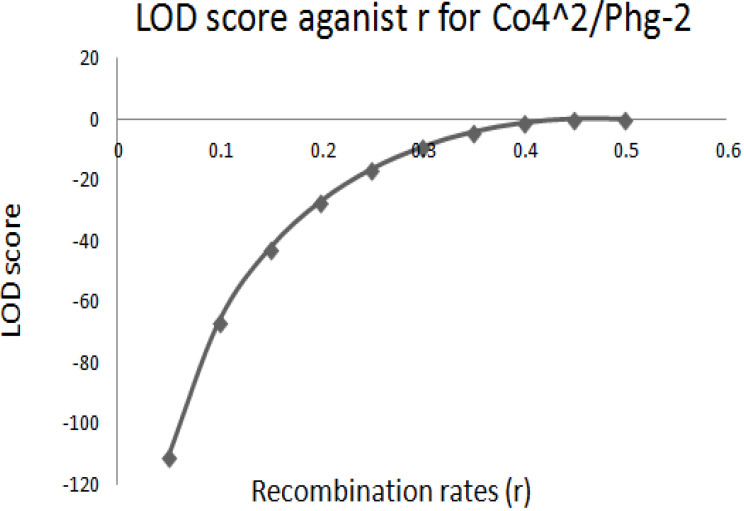
Plot of LOD scores against Recombination rates, LOD is maximum around, r = 0.47, so that is the best estimate of linkage for gene pairs *Co-4^2^/Phg-2* on bean chromosome Pv08, using BC3F6 population.

**Figure 5:**
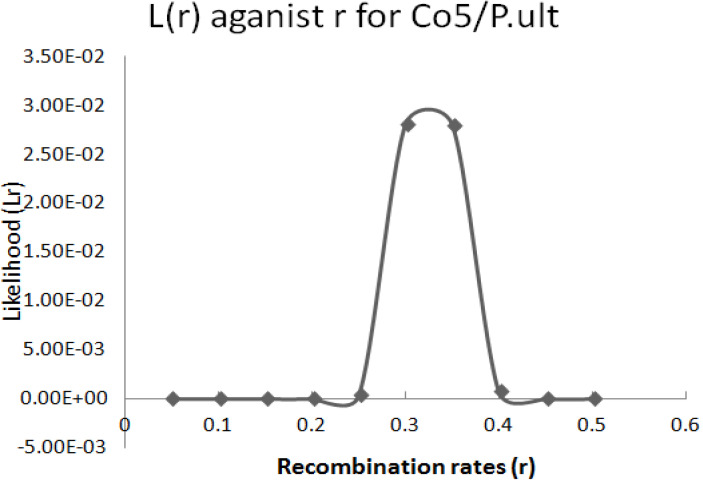
Plot of Likelihood (r) against Recombination rates, showing maximum likelihood estimate (MLE) of recombination fraction at 0.32, so that is the best estimate of linkage for gene pairs *Co-5*/ “P.ult” on bean chromosome Pv07, using BC3F6 population.

**Figure 6:**
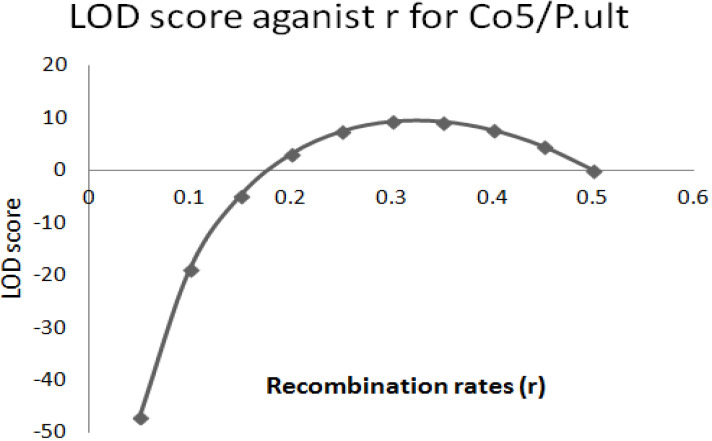
Plot of LOD scores against Recombination rates, LOD is maximum around, r = 0.32, so that is the best estimate of linkage for gene pairs *Co-5*/ “P.ult” on bean chromosome Pv07, using BC3F6 population.

The graphical presentation of likelihood (r) and maximum recombination rates at 0.47 (the maximum likelihood estimate (MLE)) extracted from Table 1 for gene pairs *Co-4^2^*/*Phg-2* are as shown in Figure 3 and 4. Similarly, the graph of the plot between LOD score from Table 1 and recombination rates ranging from 0 to 0.5 had the same pattern (Figure 4), suggesting that the recombination fraction between *Co-4^2^*/*Phg-2* was 0.47 showing weak linkage. However, the graph of the plot of likelihood (r) and recombination rates was maximum at 0.32 for gene pairs *Co-5* and “P.ult” (Figure 5). Similarly, the graph of LOD score and recombination rates (Figure 6) had the same pattern, suggesting that the recombination fraction between *Co-5* and “P.ult” was 0.32 showing a stronger linkage.

### Recombinants, likelihood and LOD scores in F2 populations

The results of likelihoods (r) and LOD scores for testing linkage among gene pairs, *Co-4^2^*/*Phg-2* and *Co-5*/“P.ult” from bean populations at F_2_ generation are shown in Table 2. For gene pairs, *Co-4^2^*/*Phg-2*, 96 genotypes were recombinants (having two genes, *Co-4^2^*/*Phg-2*) and the rest of the genotypes (n= 123) were non-recombinants (Table 2). Similarly, for gene pairs, *Co-5*/“P.ult”*, 49* genotypes were recombinants and 187 non-recombinants.

**Table 2:** Likelihoods (r) and LOD scores for testing linkage of *Co-4^2^*/*Phg-2* and *Co-5*/“P.ult” from F_2_ populations.

Loci	r	r^R^	1-r	(1-r)^N-R^	L_(r)	Lr/L(r=1/2)	LOD score [Log10(Lr/L(r=1/2))]
***Co-42/Phg-2***		**(R=96)**				**(N-R=123)**	
	0.05	1.26E-125	0.95	1.82E-03	1.98E-64	1.94E-62	-61.71
	0.10	1.00E-96	0.90	2.35E-06	2.03E-38	1.98E-36	-35.70
	0.15	8.03E-80	0.85	2.08E-09	1.44E-24	1.41E-22	-21.85
	0.20	7.92E-68	0.80	1.20E-12	8.21E-16	8.03E-14	-13.10
	0.25	1.59E-58	0.75	4.29E-16	5.89E-10	5.76E-08	-7.24
	0.30	6.36E-51	0.70	8.85E-20	4.85E-06	4.75E-04	-3.32
	0.35	1.70E-44	0.65	9.74E-24	1.43E-03	1.39E-01	-0.86
	0.40	6.28E-39	0.60	5.16E-28	0.02790	2.72856	0.44
	0.45	5.11E-34	0.55	1.16E-32	0.05108	4.99553	0.69
	0.50	1.26E-29	0.50	9.40E-38	0.01022	0.99999	-2.05E-08
***Co-5/“P.ult”***		**(R=49)**		**(N-R=187)**			
	0.05	1.78E-64	0.95	6.83E-05	1.75E-17	1.34E+03	3.13
	0.10	1.00E-49	0.90	2.78E-09	4.00E-07	3.06E+13	13.49
	0.15	4.25E-41	0.85	6.33E-14	3.88E-03	2.97E+17	17.47
	0.20	5.63E-35	0.80	7.55E-19	6.12E-02	4.69E+18	18.67
	0.25	3.16E-30	0.75	4.33E-24	0.019687051	1.51E+18	18.18
	0.30	2.39E-26	0.70	1.08E-29	0.000372339	2.85E+16	16.46
	0.35	4.56E-23	0.65	1.03E-35	6.80449E-07	5.21E+13	13.72
	0.40	3.17E-20	0.60	3.27E-42	1.49241E-10	1.14E+10	10.06
	0.45	1.02E-17	0.55	2.80E-49	4.11E-15	3.15E+05	5.49
	0.50	1.78E-15	0.50	5.10E-57	1.30E-20	1.00E+00	1.12E-06

### Graphics of likelihood, recombination rates and LOD score in F_2_ populations

**Figure 7:**
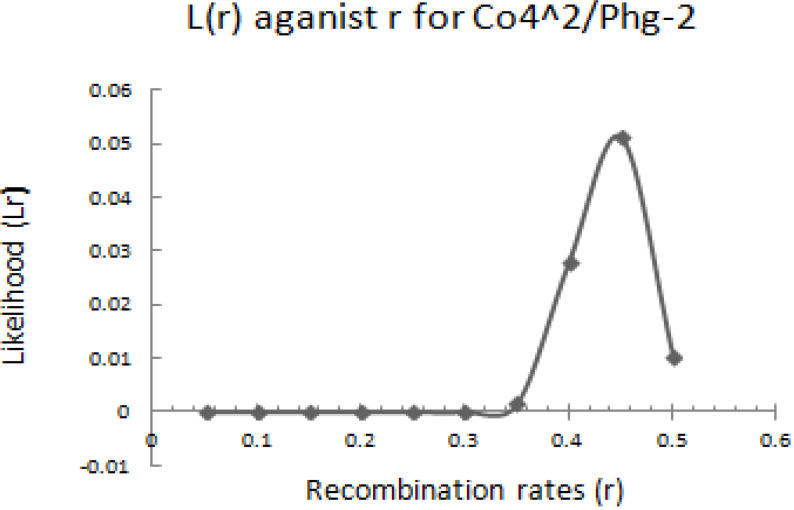
Plot of Likelihood (r) against Recombination rates, showing maximum likelihood estimate (MLE) of recombination fraction at 0.44, so that is the best estimate of linkage for gene pairs *Co-4^2^/Phg-2* on bean chromosome Pv08, using F2 population.

**Figure 8:**
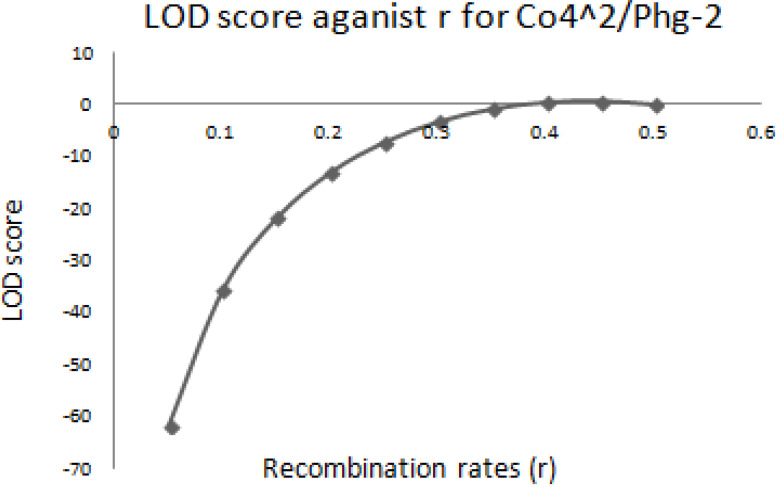
Plot of LOD scores against Recombination rates, LOD is maximum around, r = 0.44, so that is the best estimate of linkage for gene pairs *Co-4^2^/Phg-2* on bean chromosome Pv08, using F2 population.

**Figure 9:**
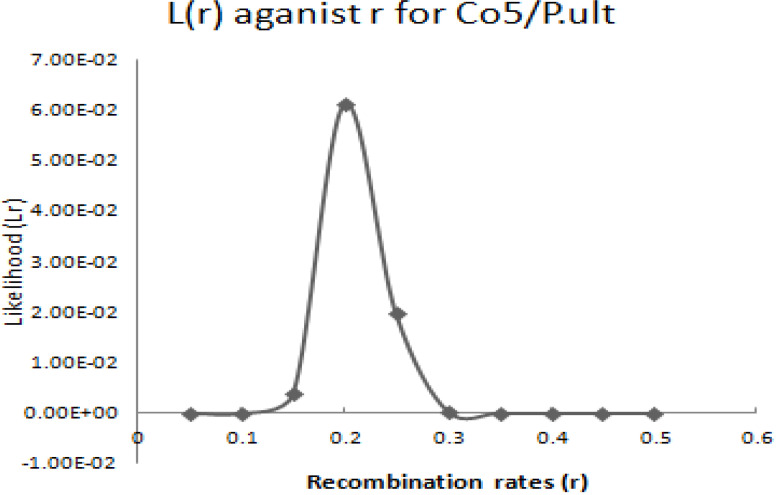
Plot of Likelihood (r) against Recombination rates, showing maximum likelihood estimate (MLE) of recombination fraction at 0.21, so that is the best estimate of linkage for gene pairs *Co-5*/“P.ult” on bean chromosome Pv07, using F2 population.

**Figure 10:**
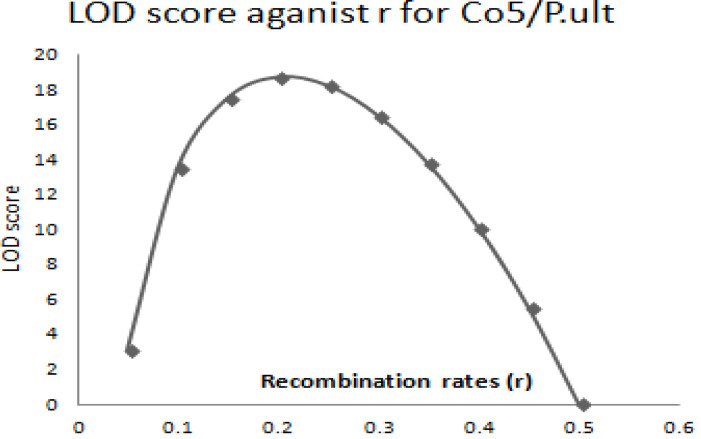
Plot of LOD scores against Recombination rates, LOD is maximum around, r = 0.21, so that is the best estimate of linkage for gene pairs Co5/“P.ult” on bean chromosome Pv07, using F2 population.

The graphical presentation of likelihood (r) and maximum recombination rates of 0.44 (the maximum likelihood estimate (MLE)) from Table 2, for gene pairs *Co-4^2^*/*Phg-2* is as shown in Figures 7 and 8. Similarly, the graph of the plot between LOD score from Table 2 and recombination rates ranging from 0 to 0.5 had the same pattern (Figure 8), suggesting that the recombination fraction between *Co-4^2^*/*Phg-2* was 0.44, showing weak linkage. However, the graph of the plot of likelihood (r) and recombination rates was maximum at 0.21 for gene pairs *Co-5* and “P.ult” (Figure 9). Similarly, the graph of LOD score and recombination rates (Figure 10) had the same pattern, suggesting that the recombination fraction between *Co-5* and “P.ult” was 0.21 showing a stronger linkage.

### Testing linkage using log-likelihood statistic

The summary of recombination fraction and likelihood data is shown in Table 3 and was used to test the significance of linkages through comparison of log- likelihood under null and alternate hypotheses (where chi value of 0.32 and 1.4 in BC_3_F_6_ and F*_2_* populations, respectively (Table 3) shows no significant difference χ^2^_tabulated_ = 3.84) for a given gene pair in both BC_3_F_6_ and F*_2_* populations. For gene pair, Co-4^2^/Phg-2 the computed between the null hypothesis (r = 0.5, no linkage) and the alternative hypothesis (r = 0.47, suggesting linkage). The marker loci for gene pairs *Co-4^2^*/*Phg-2* are thus not linked in BC_3_F_6_ and F2 bean populations studied. In contrast, for gene pairs, *Co-5*/“P.ult”*,* the computed chi value of 18.4 and 37.18 in BC_3_F_6_ and F*_2_* populations, respectively (Table 3) shows significant difference between the null hypothesis and the alternative hypothesis. The marker loci for gene pairs *Co-5*/“P.ult” are thus linked in BC_3_F_6_ and F2 bean populations studied.

**Table 3:** Comparison of log-likelihood under alternate and the null hypothesis for Co-4^2^/Phg-2 and Co-5/“P.ult” gene pairs in BC_3_F_6_ andF_2_ populations

Gene pair	Hypotheses	BC_3_F_6_			F2		

		Recombination fraction (r)	Likelihood of r, L(r)	Log of Likelihood	r	L(r)	Log of Likelihood
	H_A_	0.47	0.033	-1.48	0.44	0.051	-1.29
Co4^2^/Phg-2	H_0_	0.5	0.022	-1.64	0.5	0.010	-1.99
	2(LH_A_ - LH_0_)	-	**-**	0.32	-	**-**	1.4
*Co-5*/“P.ult”	H_A_	0.32	0.028	-1.55	0.21	6.12E-02	-1.21
	H_0_	0.5	1.77E-11	-10.75	0.5	1.30E-20	-19.8
	2(LH_A_ - LH_0_)	-	**-**	18.4*	-		37.18*

*Alternative hypothesis (*H_A_*) is when *r* is estimated at its MLE and the null hypothesis (H_0_) is when r is fixed at 0.5. 2(LH_A_-LH_0_)* shows that the difference between the log-likelihood multiplied by a factor of 2 for technical reasons, so that this quantity will be distributed as the familiar chi (_χ_^2^) statistic. Critical value for test-statistics at 1% level of significance is 3.84. Asteric (*) of log-likelihood shows significant difference between recombination fraction (r) values at H_A_ versus H_o_ suggesting linkage between corresponding gene pairs.

## DISCUSSION

The objective of this study was to estimate recombination fractions and genetic linkage between gene pairs, *Co4^2^/Phg-2* on bean chromosome Pv08 and *Co-5*/“P.ult” on chromosome Pv07. A strong genetic linkage among a pair of molecular markers located less than five centi Morgans (cM) apart on common bean chromosome implies that their two linked genes could be selected with only one marker to reduce genotyping costs.

The results show incomplete genetic linkage between gene pairs, *Co-4^2^/Phg-2* and *Co-5*/“P.ult” on common bean chromosomes Pv08 and Pv07, respectively.The recombination fraction summarized in Table 3 are indicators of the degree of linkage and was higher in BC_3_F_6_ than F_2_ populationsfor gene pairs,*Co-4^2^/Phg-2* (47cM vs 44 cM) and *Co-5*/“P.ult” (32 cM vs. 21 cM). This difference was attributed to the two generation studied with significant differences in levels of genetic variations.

As reported under population development under the materials and methods, the F_2_ populations derived their parents from from progenies of the BC_3_F_6_ population with single genes targerted. At BC_3_F_6_ the bean populations had possibly accumulated more recombinations than in F_2s._ Secondly, theprogeny lines in BC_3_F_6_ genotyped were derived from a four way cross comprising four parents used to develop the genetic pyramids (Figure 2) with a high genetic diversity and population structure (Okii et al., 2017).

The hypothesis that physically linked genes for bean diseases, for example anthracnose (*Co-4^2^*) and angular leaf spot resistance (*Phg-2*) located on bean chromosome Pv08 co-segregate in bean populations due to genetic linkages was tested in this study. However, the weak genetic linkage between marker pairs studied shows that each of the four genes mentioned earlier have to be tagged with a corresponding linked markers during MAS. The study aimed to suggest strategies for reducing population size during gene pyramiding by finding a single marker locus (position of the chromosome) between genes or markers for simultaneous selection of resistance genes on the same bean chromosome(s), with linkages in coupling (Staub et al., 1996). Theoretical investigations that probe the potential of MAS are, however, of practical importance (Staub et al., 1996).

In other studies on common bean, maximum linkage with no recombinants (0.0 cM) was reported for gene pairs, *Co-1^4^* and *Phg-1* for anthracnose and angular leaf spot diseases in bean cultivar AND277 (GonçalvesVidigal et al., 2011) and suggested overlap or very tight linkage of *Co-1^4^* and *Phg-1* loci in the bean genome. The large genetic separation of SCAR markers SBB14 for *Co-4^2^* and SN02 for *Phg-2* corroborates their physical positions in the Andean bean reference cultivar G19833 (Schmutz et al., 2014), with SBB14 situated at 2,758,731 base pairs (Burt et al., 2015) and SN02 at 58,535,51758,536,216 on Pv08 (http://phaseolusgenes.bioinformatics.ucdavis.edu/).

To define whether two markers are in linkage is to test whether the recombination fraction between these two markers is less than 0.5 (Balding et al., 2007). This hypothesis testing problem can be carried out using the likelihood ratio test Ott et al, 2015; Balding et al., 2007). Similarly, Sun et al. (2012) proposed recombination frequencies of 15 cM as the threshold for strong linkages among loci, while LOD scores above 3 indicate strong genetic linkages (Ott et al., 2015). The threshold genetic distance of 5 cM was recommended as strong indicator of linkage between molecular markers and resistance genes during MAS (Collard and Mackill, 2008).

Therefore, the approaches used in this study contributed to two loci linkage mapping techniques in segregating plant populations through genotyping with markers. However, in situations where computer programs are used to integrate phenotypic and genotypic data sets, estimated LOD scores and likelihood values provide a threshold value for testing genetic linkage (Churchill and Doerge, 1994).

Key statistical methods used in the study are reported by Geffroy et al., (2008) to show possible ways of reducing the number of laboratory samples screened with markers to reduce genotyping costs while still improving the efficiency of MAS for traits influenced by few genes such as diseases in common bean. The results should complement other useful genetic maps developed earlier for improving common bean for combining resistance to several diseases and quantitative trait loci (QTL) of economic importance (Kelly et al., 2003).

This study was based on dominant SCAR markers, which cannot differentiate homozygotes progenies from heterozygotes, we therefore recommend use of co-dominant markers to estimate linkage among gene pairs; *Co-4^2^/Phg-2, Co-5/*“P.ult” in early generations such as F_2_s and F_3_s using a moderate population size (of 50 individuals) proposed by Sun et al., (2012). The following are other recommendations: 1) sequencing SCAR markers which are strongly linked to targeted resistance genes in parental cultivars, then annotate chromosomal regions flanking the markers to find alternative markers and potential candidate genes; 2) Phenotyping the bean populations with pathogens in addition to genotyping and establish the correlations; and 3) Reciprocal crosses can be used to test the effect of maternal effects on linkage estimates among gene pairs or markers.

## CONCLUSION

There was weak linkage among gene pair, *Co-4^2^/Phg-2* on bean chromosome eight. The linkage was however relatively stronger among gene pair, *Co-5/*“P.ult”. There was difference in the value of recombination fraction between the BC_3_F_6_ and F_2_ population. This implies that selection for each of the resistance genes, *Co-4^2^, Phg-2, Co-5* and “P.ult” requires to be selected with their own SCAR marker due to lack of strong genetic linkages among these genes during marker assisted gene pyramiding targerting all the four genes in the same background.
